# A Hidden Markov Model reveals magnetoencephalography spectral frequency-specific abnormalities of brain state power and phase-coupling in neuropathic pain

**DOI:** 10.1038/s42003-022-03967-9

**Published:** 2022-09-21

**Authors:** Camille Fauchon, Junseok A. Kim, Rima El-Sayed, Natalie R. Osborne, Anton Rogachov, Joshua C. Cheng, Kasey S. Hemington, Rachael L. Bosma, Benjamin T. Dunkley, Jiwon Oh, Anuj Bhatia, Robert D. Inman, Karen Deborah Davis

**Affiliations:** 1 Division of Brain, Imaging, and Behaviour, Krembil Brain Institute, University Health Network, Toronto, ON M5T 2S8 Canada; 2 Institute of Medical Science, University of Toronto, Toronto, ON M5S 1A8 Canada; 3 Neurosciences & Mental Health Program, The Hospital for Sick Children Research Institute, Toronto, ON M5G 0A4 Canada; 4 Diagnostic Imaging, The Hospital for Sick Children, Toronto, ON M5G 0A4 Canada; 5 Department of Medical Imaging, University of Toronto, Toronto, ON M5T 1W7 Canada; 6 Div of Neurology, Dept of Medicine, St. Michael’s Hospital, Toronto, ON M5B 1W8 Canada; 7 Department of Anesthesia and Pain Medicine, Toronto Western Hospital, and University of Toronto, Toronto, ON M5T 2S8 Canada; 8 Division of Immunology, University of Toronto, Toronto, ON M5S 1A8 Canada; 9 Department of Surgery, University of Toronto, Toronto, ON M5T 1P5 Canada

**Keywords:** Neural circuits, Neuropathic pain, Chronic pain

## Abstract

Neuronal populations in the brain are engaged in a temporally coordinated manner at rest. Here we show that spontaneous transitions between large-scale resting-state networks are altered in chronic neuropathic pain. We applied an approach based on the Hidden Markov Model to magnetoencephalography data to describe how the brain moves from one activity state to another. This identified 12 fast transient (~80 ms) brain states including the sensorimotor, ascending nociceptive pathway, salience, visual, and default mode networks. Compared to healthy controls, we found that people with neuropathic pain exhibited abnormal alpha power in the right ascending nociceptive pathway state, but higher power and coherence in the sensorimotor network state in the beta band, and shorter time intervals between visits of the sensorimotor network, indicating more active time in this state. Conversely, the neuropathic pain group showed lower coherence and spent less time in the frontal attentional state. Therefore, this study reveals a temporal imbalance and dysregulation of spectral frequency-specific brain microstates in patients with neuropathic pain. These findings can potentially impact the development of a mechanism-based therapeutic approach by identifying brain targets to stimulate using neuromodulation to modify abnormal activity and to restore effective neuronal synchrony between brain states.

## Abstract (web summary)

A comparative analysis on resting-state magnetoencephalography data from healthy controls and patients with chronic pain reveals spectral frequency-specific dysregulation and a temporal imbalance of brain microstates in chronic neuropathic pain.

## Introduction

Neuropathic pain is a major healthcare challenge due to limited efficacious therapies, and it is a tremendous burden to individuals and the society^1^. Neuropathic pain is characteristically experienced as intense pain with unpleasant characteristics such as excruciating shooting, stabbing, or burning, and can be stimulus-evoked pain and also involve sensory loss^2^. The alterations in brain activity in patients with central and peripheral neuropathic pain are not well-defined, but growing evidence suggest impairment of the dynamic organization of neural network activity; considered to be brain microstates.

Converging findings indicate that neuropathic pain is associated with abnormal brain structure and function^3^. Functional magnetic resonance imaging has identified dysfunctions of brain networks within the dynamic pain connectome (DPC)^4^ in several chronic pain conditions that have a neuropathic pain component such as multiple sclerosis^5^, ankylosing spondylitis^6–8^, chronic low back pain^9^, and other neuropathic etiologies^10,11^. Moreover, technologies that can examine brain activity at millisecond resolution (i.e., electroencephalography and magnetoencephalography (MEG)) have reported that people with chronic pain exhibit altered neuronal oscillations at specific frequency bands^12,13^, such as an increase in resting-state alpha-band (8–13 Hz) spectral power^13–17^ and slower peak alpha frequency^14,17–19^; some of which are ameliorated after successful pain treatment^20,21^. Our group and others have identified that alpha-band abnormalities in people with chronic pain were more likely to occur if individuals had neuropathic pain attributes^14,15^. Given these and other findings, alpha oscillations have been proposed as a potential neuromarker of acute pain sensitivity^22^ and neuropathic pain^3^. There is also evidence for the involvement of local neuronal activity and global functional connectivity dysfunction in the other frequency bands, such as theta (4–8 Hz), beta (13–30 Hz), and low gamma (30–60 Hz) frequencies^23,24^ in all type of pain (e.g., neuropathic, nociceptive, nociplastic).

Disruptions in oscillating signals measured on the surface of the scalp reflect dysrhythmic activity of neurons in the network, which influence the effectiveness of neuronal coordination between brain regions (akin to a bug in the neural code)^25,26^. This is grounded in the concept that coherently oscillating neuronal groups interact with greater efficiency^27^, and so such a disruption could impact sensory and cognitive response to pathological states^28^, including pain. Another fundamental neural mechanism is phase-coupling or phase synchronization which refers to the relationship between oscillation phases in different brain regions^29^. Intrinsic resting-state brain networks have spectral characteristics such that their activity are most pronounced in one frequency band, but could also exhibit spontaneous fast changing phase-coupling activity and this is thought to be an important contribution to cognitive processes by regulating neuronal communication^30^.

The Hidden Markov Model (HMM)^31,32^ approach is used to reduce cortical activity at rest into sequences of transient, intermittently reoccurring events, known as brain states. At each brain state, large-scale networks are characterized by specific patterns of power and phase-coupling, and these are factorized as a function of frequency (i.e., spectrally resolved)^33–35^. This approach applied to neuroimaging data was successfully used to characterize profiles of time-varying connectivity associated with opioid analgesia^36,37^. Using MEG data, this method provided a fine probabilistic estimation of when the different brain states are active^32,38^. Thus the HMM approach can be used to reflect the spontaneous micro-temporal brain dynamics at rest associated with chronic pain conditions, given a choice of free parameters and prior distributions. It remains unknown, however, whether and how brain states are spatially, spectrally, and temporally altered in neuropathic pain.

The aim of the present study was to determine whether there are MEG brain microstates dysfunction associated with different chronic pain conditions with a neuropathic character. To this end, we applied an HMM statistical approach to examine resting-state MEG data from 40 patients with central and peripheral chronic neuropathic pain compared to 40 age- and sex-matched HCs. MEG data were source-reconstructed to 36 dipoles covering the entire DPC. Twelve-states HMM solution identified brain microstates for networks of the DPC^4^ including the default mode (DMN), salience (SN), ascending nociceptive pathway (ANP), sensorimotor network (SMN), and other higher-order cognitive networks. A prominent finding was that there were distinct temporal and spectral properties abnormalities in the neuropathic pain group that were characterized by higher alpha power in the brain state identified for the right ANP network, and higher beta power in the SMN. Also, in participants with neuropathic pain compared with healthy controls (HCs), the interval of time between transitions to (i.e., visits of) the SMN was shorter, and their brain spent more active time in this state. These correlational neuromarkers indicate that neuropathic pain may impair the dynamic coordination of neural network activity. Our findings provide support for using HMM to understand the organizational framework underlying neural synchrony and detect the functional consequences of neuropathic pain.

## Results

### Identification of 12 brain states using HMM in healthy individuals and those with neuropathic pain

**Fig. 1:**
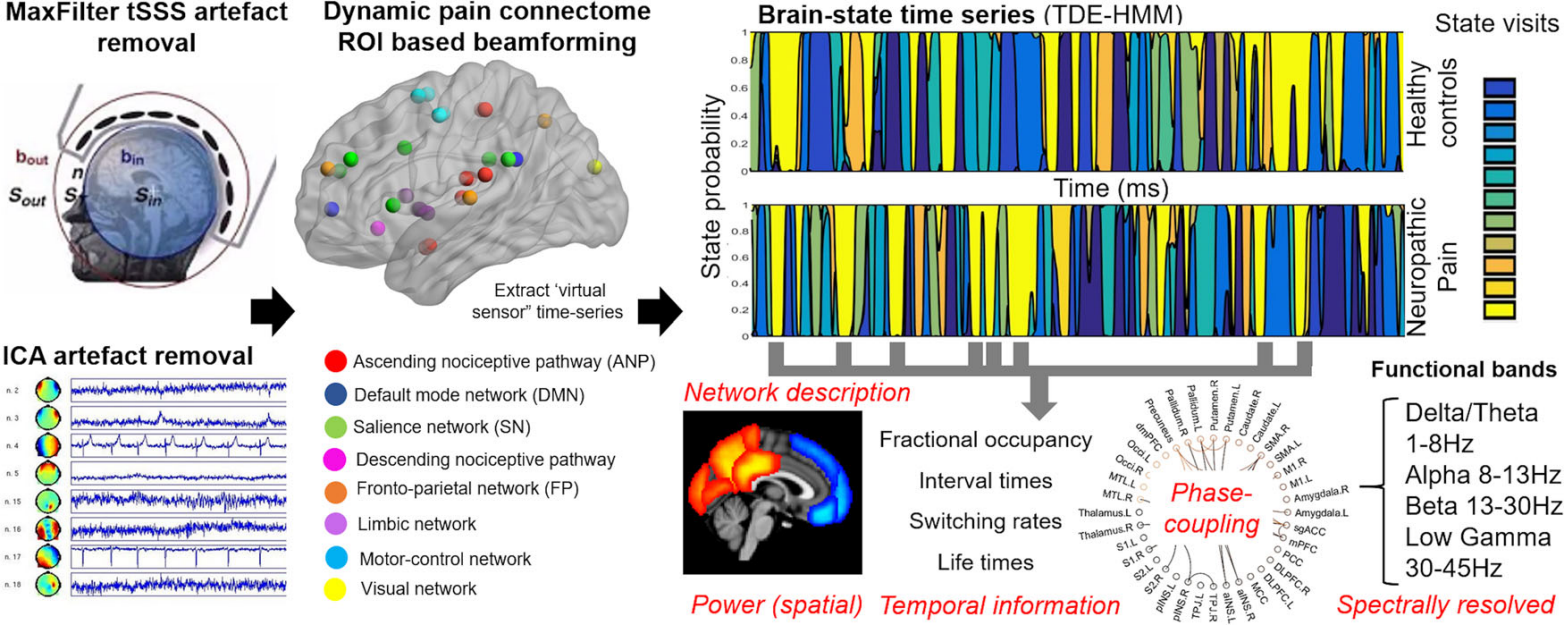
Illustration of time-delay embedded Hidden Markov Model (TDE-HMM) approach applied on MEG data. MEG data preprocessing (Independent component analysis (ICA) for artifact removal, source reconstruction, parcellation, leakage correction, and sign disambiguation—see Methods). We used a linearly constrained minimum variance beamformer to extract a continuous time series for nodes of the dynamic pain connectome based on anatomical 3D T1 scans of each participant. Spontaneous cortical activity transiently organizes into frequency-specific phase-coupling networks. Principal component analysis was applied for dimensionality reduction, and HMM inference was then used to identify the state time-courses (state probability) and the state parameters in HCs and those with neuropathic pain. Each state was characterized as having its own distinct spatial, temporal, and spectral properties. Individual power maps and phase-coupling patterns (networks) were estimated for active states and illustrated by their temporal features (i.e., fractional occupancy, interval times, switching rates, and lifetimes). States were spatially defined and spectrally resolved according to the main frequency bands (i.e., delta/theta, alpha, beta, and low gamma).

We used concatenated MEG resting-state data from 40 participants with neuropathic pain (20 females and 20 males) and age/sex-matched healthy subjects to map to a 36-region parcellation of the DPC^4,14,15,39^ using the time-delay embedded HMM methods developed by Vidaurre et al^32,34^ (see methods). A central and free parameter in HMM inference is the number of states, which must be chosen before further evaluation. Here, we identified 12 HMM states in both the chronic pain and HC groups (Fig. 1).

The HMM method is a mathematical framework used to identify recurring states in brain functional data^33^. This allows for the identification of brain-wide networks characterized by specific patterns of power and phase-coupling connectivity, which are spectrally resolved (i.e., power and phase-coupling modes that are defined as a function of frequency). These patterns are also temporally resolved, meaning that the method provides a probabilistic estimation of when the different networks are active, according to HMM assumptions. Importantly, while the spatial and spectral description of the states is common to all subjects, each subject has their own state time-course that represents the probability of each HMM state being active at each instant.

We computed the HMM for 6 and then 12 states. The optimal number of states is a compromise to select the highest number of states while still having a reliable solution (see methods). Each state of activity can be further partitioned into a set of functional sub-states. The six-states solution provided state information that was consistent with the 12-states solution except it failed to convey relevant information about the separation of some network states (see Supplementary Fig. 1). Some states from the original 12-states analysis were fused into fewer states.

The HMM state distributions are patterns of covariance across channels and time points within a certain window (i.e., channels X time points by channels X time points). Principal component analysis is used to reduce the size of the state covariance matrices and aims to explain the highest possible amount of variance in the time series (see Methods). This reduction explained on average 68% of variance (lowest and highest values across subjects were 61% and 72%, respectively).

### Description of brain states in healthy individuals and in neuropathic pain

Spatial maps of power and phase-coupling connectivity were each averaged across a wideband frequency range (1–30 Hz) to describe the overall characteristics of each state.

We determined the regional power and functional connections that are significantly stronger for one state compared to the other states. To do this, we conducted nonparametric statistical testing on the between-subject variability calculated from each individual’s spectral information (i.e., power and connectivity; see methods).

**Fig. 2:**
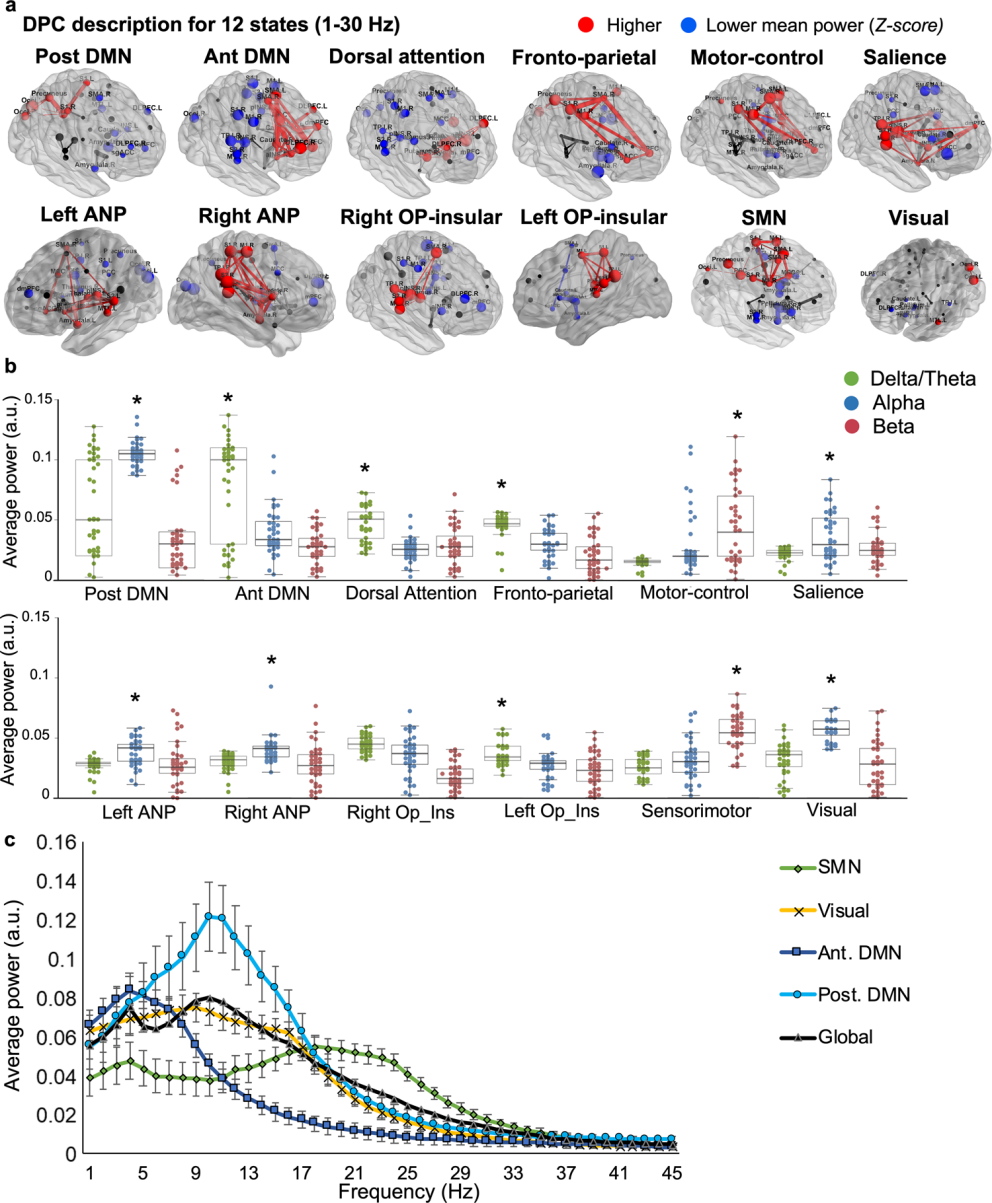
Brain states identified using an HMM show functional networks of spectral power and coherence in the DPC. **a** Brain regions with significant power and phase-coupling are represented across a wideband frequency range (1–30 Hz) for the 12 estimated states in *n* = 40 healthy controls. Node spectral power is relative to the temporal average, and node size is in relation to the mean power (*Z* score) across states (blue and red colors reflect power that is lower or higher than the average over states, respectively). Edges between nodes show functional connectivity, and only significant and high-valued connections are shown. Significant coherences were mainly found between active nodes in each state. The same 12 states were found in the patients cohort, and the nodes’ repartition was similar across a wideband frequency range. **b** Spectral power averaged across all brain regions in brain states as a function of frequency (green: delta/theta, blue: alpha, and red: beta). **c** Spectral profiles of the DMN (post. DMN: blue; ant. DMN: purple), SMN (green), and visual (yellow) states, in terms of power averaged across brain regions, in comparison to the grand average (black line); Standard deviations are represented. *P < 0.001; permutation *P* value, 5000 bootstrap samples. DMN Default mode network, ANP ascending nociceptive pathway, OP-insular (operculo-insular), SMN sensorimotor, DPC dynamic pain connectome.

This yielded power maps of the brain regions that are associated with statistically significant wideband spectral power and connectivity in each state in both groups at rest. The pattern of power activity and functional coherence in each state was bilateral, in seven states but was lateralized to one hemisphere in the other five states. The brain states in healthy individuals and those with neuropathic pain corresponded to commonly observed intrinsic resting-state brain networks in the DPC^6,40^, but since a 12-state solution is used in the model, some high-order networks are divided in subnetworks. The brain states identified in both the HC and neuropathic pain groups included: (1) the posterior and anterior part of the DMN (post DMN, ant DMN); (3) Dorsal/limbic attention; (4) fronto-parietal (FP); (5) motor-control; (6) SN; (7) left and (8) right ascending nociceptive (i.e., sensory) pathway networks (left and right ANP); (9) left and (10) right operculo-insular networks (OP-insular); (11) sensorimotor (SMN), and (12) visual networks (also see Supplementary Tables 1 and 2). Nodes showing significant power in each state tend to accompany increases in phase-locking. Figure 2a shows large-scale brain microstates organization (power and phase-coupling) in HCs (i.e., the basal condition).

### State-specific frequency bands

We next examined the state-specific frequency bands. To do this, we factorized the frequency bins (1–45 Hz) into four frequency bands using a data-driven frequency decomposition^32^. We used non-negative matrix factorization as the decomposing method. The approach identified four modes corresponding roughly to the classical frequency bands: Delta/theta (from 2 to 8 Hz), alpha (from 8 to 14 Hz), beta (from 14 to 30 Hz), and low gamma (from 30 to 45 Hz).

As in Vidaurre et al.^32^, strong state-specific differences in the gamma band could not be observed with this approach due to the relatively low signal-to-noise ratio in higher-frequency bands, and therefore, we only show results for the delta/theta, alpha, and beta bands.

The average power across brain regions as a function of frequency is shown in Fig. 2b. Some states have their power more represented in one frequency band. In general, there was variability in the dominant oscillation frequencies across states in the DPC (*P*_corrected_ < 0.001; permutation tests). We found that theta/delta frequency bands were dominant oscillations in frontal states (involved in cognitive functions) including the frontal DMN, the dorsal attention, the FP and the operculo-insular states. Alpha was largely limited to parietal and occipital regions, and is dominant in the post DMN, the visual, the ANP states and to a lesser extent the SN state (alpha/beta). The beta band was prominent in the SMN and motor-control states.

In summary Fig. 2c, in both groups, the posterior DMN state was characterized by strong power and coherence within the alpha frequency band (dominant peak frequency ≈12 hz), and was a dominant state compared to the others in term of power, whereas the frontal DMN state was dominated by the delta/theta frequency band (≈5 hz). The SMN state showed stronger power in the beta band (≈20 hz), and the visual state was more represented in the alpha band (≈9 hz).

### Differences in spectral power and functional coherence in healthy individuals and those with neuropathic pain

We next examined the spatial distribution changes of power and connectivity between neuropathic pain and HC groups using the three previous frequency bands (i.e., delta/theta, alpha, beta bands).

### Spectral power

**Fig. 3:**
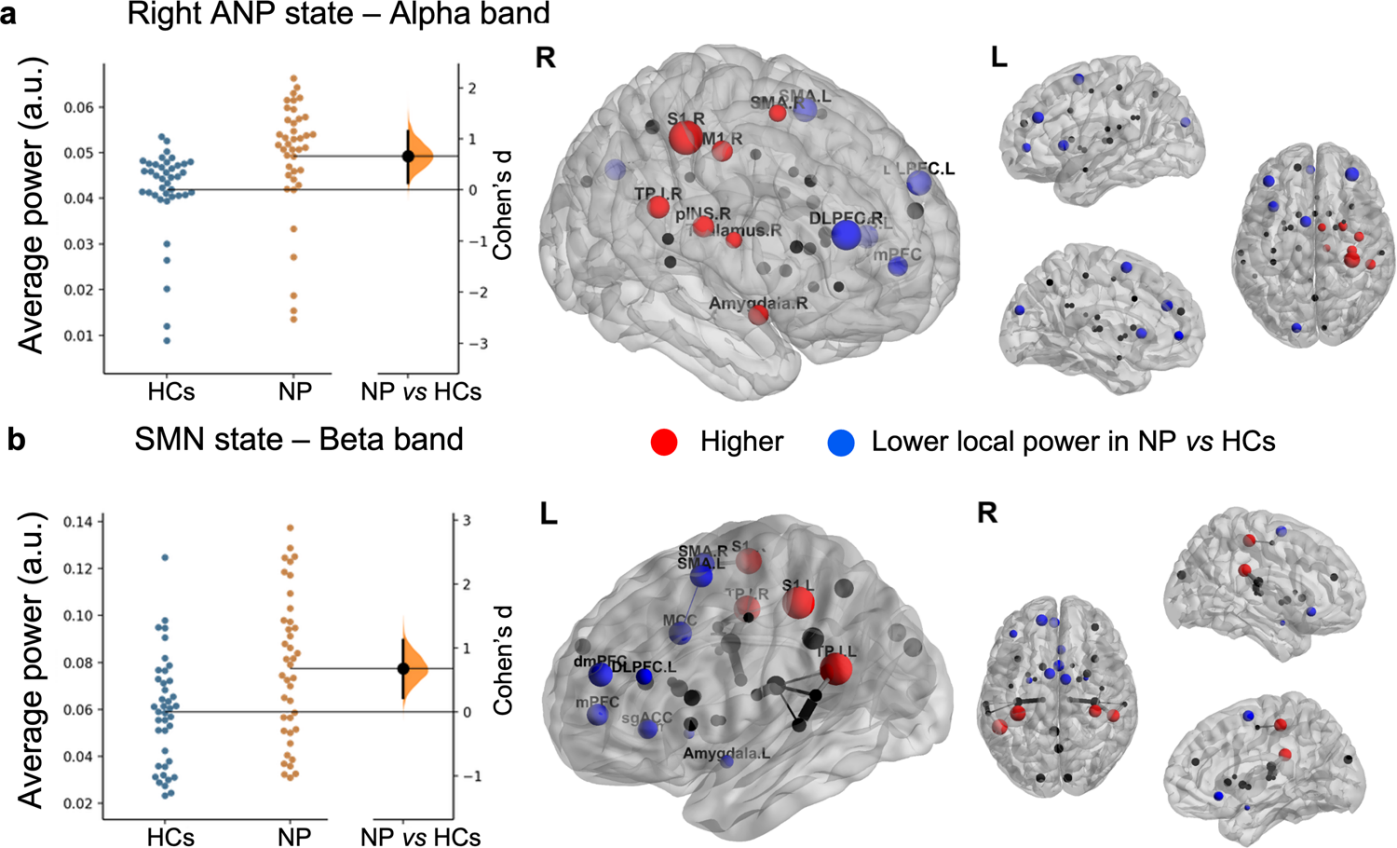
Individuals with neuropathic pain had higher alpha power in the right ANP state and higher beta band power in the SMN state. Brain nodes in neuropathic pain (NP) showing significant local power increased (red nodes) and decreased (blue nodes) in the **a** right Ascending nociceptive pathway (ANP: higher alpha power in the right thalamus, primary somatosensory (S1), and motor cortex (M1), posterior insula (pINS), temporo-parietal junction (TPJ) and lower power in the dorsolateral prefrontal cortex (DLPFC), medial prefrontal cortex (mPFC), and left anterior insula (aINS) and **b** sensorimotor (SMN: S1 and TPJ, but had lower power in frontal regions including the DLPFC, mPFC, dorso-medial prefrontal cortex (dmPFC), subgenual anterior cingulate cortex (sgACC), midcingulate cortex (MCC) and sensorimotor area (SMA) state compared with healthy controls (HCs) are depicted. The effect size (Cohen’s *d*) between the average power of *n* = 40 healthy controls and *n* = 40 individuals with neuropathic pain is shown in the Gardner-Altman estimation plots. 5000 bootstrap samples were taken; the confidence interval is bias-corrected and accelerated. Each dot on the brain maps represents a brain region, and its size is in relation to the difference in spectral power between the neuropathic pain and control groups (i.e., proportional to the effect size, larger dot means a stronger difference).

We first examined whether there were abnormalities in spectral power in the patients with neuropathic pain. We found that the neuropathic pain cohort exhibited higher spectral power compared to the HC group in two instances: (1) at alpha frequency in the right ANP (Cohen’s *d* = 0.662 [95.0%CI 0.137, 1.15]; *p* = 0.0044 two-sided permutation *t* test, 5000 bootstrap samples were taken; the confidence interval is bias-corrected and accelerated), and (2) at beta frequency in the SMN state (Cohen’s *d* = 0.678 [95.0%CI 0.217, 1.12]; *p* = 0.004) compared to HCs (Fig. 3a). The averaged power did not change in the other HMM states and frequency bands in neuropathic pain compared with HC.

We next conducted a permutation analysis using network-based statistic^41,42^ to identify local nodal changes of power in these two brain states (SMN and right ANP) that were found to be abnormal in neuropathic pain. This analysis indicated that at the nodal level in the right ANP state, the neuropathic pain group had higher alpha power in the right thalamus, primary somatosensory cortex (S1), posterior insula (pINS), temporo-parietal junction (TPJ), primary motor cortex (M1) and lower power in the dorsolateral and prefrontal cortex (DLPFC), mid PFC (mPFC), and left anterior insula (aINS) compared with HCs (*p* < 0.01, corrected 5000 permutations, NBS method). In the SMN state, the neuropathic pain group exhibited stronger beta power bilaterally in the S1 and TPJ but had lower power in frontal regions including the DLPFC, mPFC, dmPFC, subgenual anterior cingulate cortex (sgACC), midcingulate cortex (MCC) and somatosensory-motor area compared to HCs (Fig. 3 and Supplementary Table 3).

### Abnormal functional coherence in neuropathic pain

**Fig. 4:**
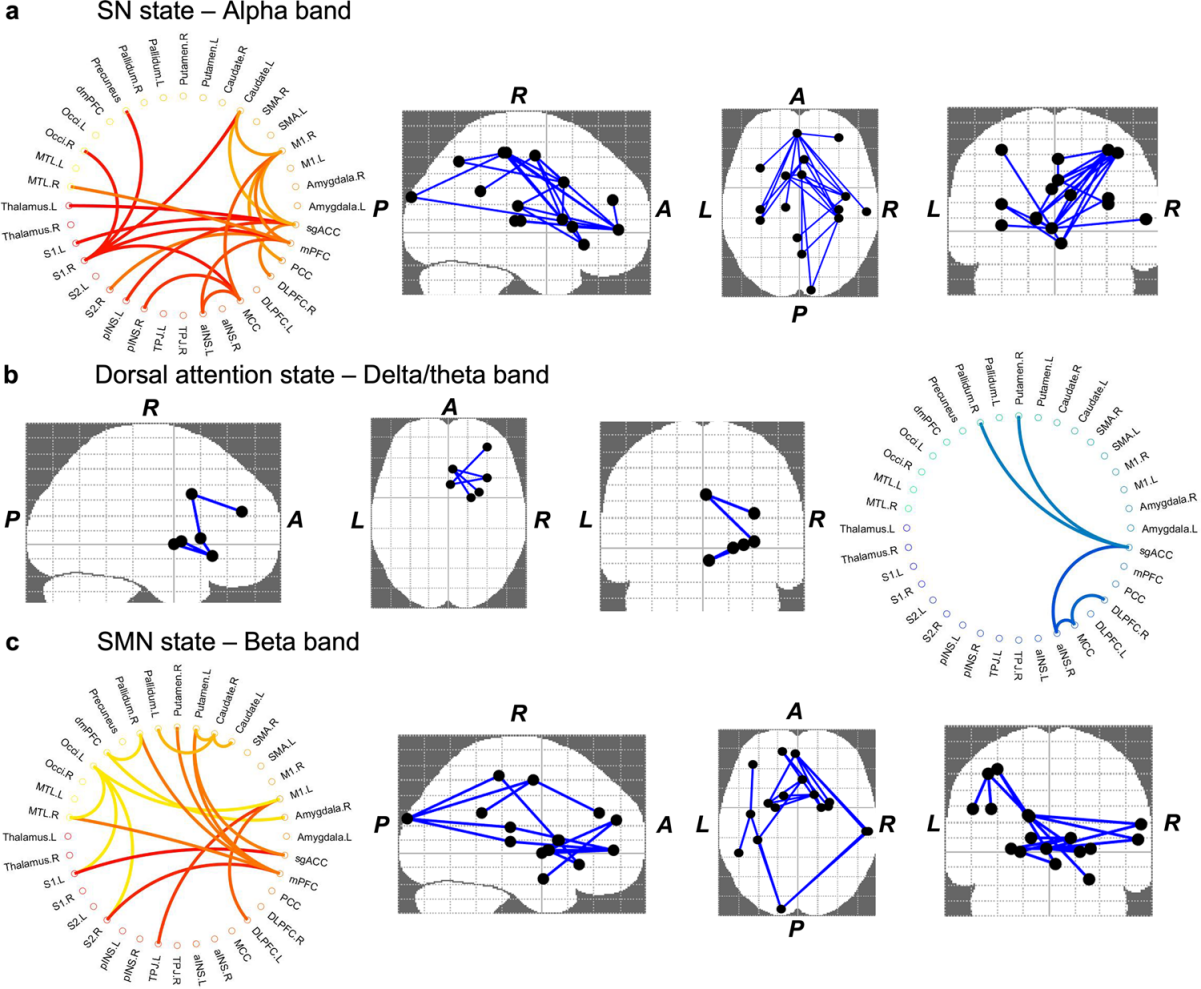
Coherence changes in individuals with neuropathic pain compared to HC group in specific frequency ranges. The brain maps show connections (in blue) associated with significant changes between neuropathic pain and healthy control groups in one brain state; each dot (black) represents one brain region. In the circular coherence plots, blue and red/yellow colors reflect respectively coherence that is lower and higher in neuropathic pain than in the control group. In neuropathic pain patients, we found that the coherence was **a** higher in the salience (SN; left: S1, pINS, aINS, thalamus, caudate, mPFC, MCC, sgACC; right: DLPFC, M1, medial temporal lobe (MTL), pINS, S1, posterior cingulate cortex (PCC), Precuneus, occipital lobe) state in the alpha band; and **b** in the sensorimotor (SMN; dmPFC, mPFC, sgACC, MTL, pallidum, putamen, caudate Left: occi, TPJ, S1, M1, DLPFC, right: amygdala, S2) state in the beta band, **c** whereas coherence was lower in the dorsal attention state (sgACC, MCC; right: aINS, DLPFC, pallidum, putamen) in the delta/theta compared with healthy control. *P < 0.01, corrected 5000 permutations, network-based statistic method.

The degree of synchronization between regions of the brain can be tested directly using MEG functional connectivity assessment (i.e., functional coupling), which can reflect certain aspects of inter-regional neuronal communication driven by common cognitive functions (i.e., cognitive complexity, see^43,44^). For MEG this tends to mean assessment of phase synchrony between oscillations in frequency bands of interest. We determined the phase-coupling for both the neuropathic pain and HC groups. We found that in some states, there are functional coherence abnormalities related-to neuropathic pain at particular frequency bands (*p* < 0.01, corrected 5000 permutations, NBS method). Specifically, when comparing the neuropathic pain group to the HCs, we found that the connectivity was higher in the SN state between bilateral brain regions including the aINS, MCC, DLPFC, mPFC, sgACC, posterior cingulate cortex (PCC), M1, S1, and subcortical nodes in the alpha band, and was also stronger in the SMN state between the DLPFC, PFC, sgACC, medial temporal lobe (MTL) and subcortical regions in the beta band. In contrast, we found that the functional coherence was lower in the dorsal attention state between the sgACC, right aI, DLPFC, and limbic regions in the delta/theta band (Fig. 4; see results details in Supplementary Table 3).

These findings thus indicate that, in neuropathic pain, there are atypical spatial distribution of power and connectivity in the brain and that these brain state changes are characterized by specific temporal features.

### Temporal features of the brain functional states

The HMM inference allows us to estimate the time course of the visits to each of the brain states, which provides insight into the amount of time spent in a state before moving into a new state. We used several indices to represent the states’ temporal characteristics (i.e., fractional occupancy, interval times, switching rate, transition probabilities, and life time, see Methods). These are not biological measures and depends on prior HMM hyperparameters such as the distribution of the transition probability matrix. Thus, we examined only relative differences between the states and between the chronic pain and HC groups.

**Fig. 5:**
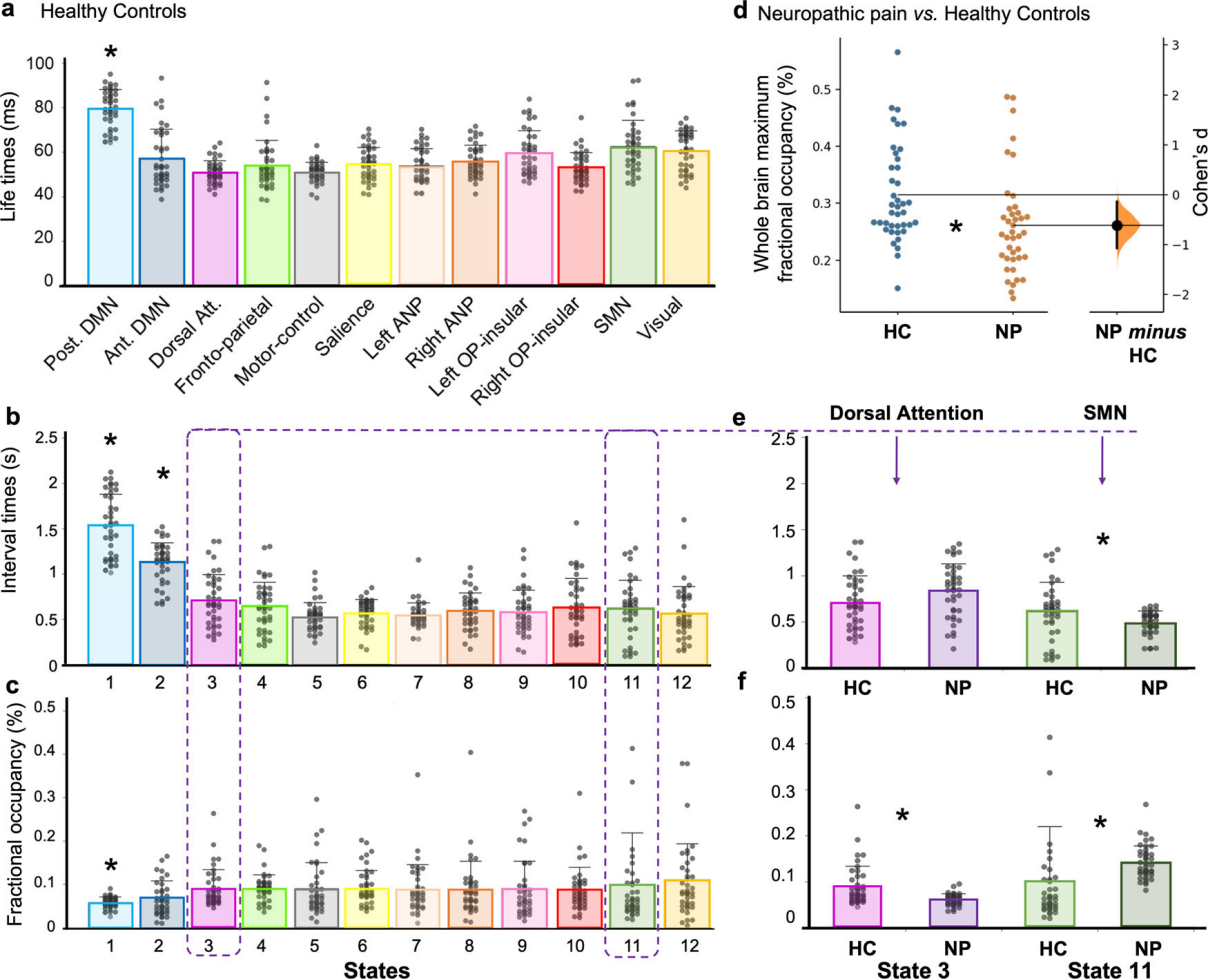
Brain states temporal features: chronnectome in healthy controls and disturbances associated with neuropathic pain. **a** Distribution of state life times, **b** distribution of interval times between state visits, and **c** fractional occupancies (proportion of time spent in each state) are depicted for the *n* = 40 healthy controls group. The two states related to the default mode network (DMN) have distinct temporal features compared with the other states. The posterior DMN state had higher lifetimes, and both posterior and anterior DMN states had higher interval times, but the proportion of time spent in the posterior DMN state was lower compared to the other states (*P* < 0.01). In *n* = 40 participants with neuropathic pain, **d** the maximum fractional occupancy was lower, and **e** the sensorimotor (SMN) was more frequently active compared with *n* = 40 healthy controls. **f** The proportion of time spent was higher in the SMN state and lower in the dorsal attention state compared to healthy controls. Standard deviations are shown. *P < 0.01; permutation *P* value, 5000 bootstrap samples. DMN Default mode network, SMN sensorimotor, HC healthy control, NP neuropathic pain.

In both the healthy and pain groups, all HMM states were on average short-lived, with their life times typically lasting from 50–80 ms. The posterior DMN state had higher life times than the other states in both groups. In addition, the two states related-to the DMN (posterior and anterior) had higher interval times (Fig. 5a). Thus, the visits of these two high order cognitive states were temporally distinct compared to the other states (permutation testing, *P* < 0.001 for both states). This was especially pronounced for the posterior DMN state (Fig. 5b). These results are consistent with previous works^32^, however in our results the fractional occupancy (i.e., reflecting the proportion of time spent in each state) of the post DMN state was lower than other states (Fig. 5c; permutation testing, *P* < 0.01). Overall, our findings demonstrated that the DMN states last longer (especially the posterior part) but are not revisited for longer periods and tend to be visited less frequently than other states such as the visual or SMN states.

We further found that state switching was a fast dynamic phenomenon in terms of power and phase-locking information. Specifically, we found states switched on average approximately every 40 to 100 ms. The maximum fractional occupancy was computed per subject and indicated that in all subjects, there was a mixture of states and no single state was dominant (see Fig. 5d). However, the state distribution was quite homogenous across subjects in both groups, although in some subjects there was higher representation of some states than others. This inter-individual variability demonstrates that subjects have different degrees of state representation, possibly related to specific subject traits.

### Temporal abnormalities (i.e., time spent within or between states) in neuropathic pain

We found that the maximum fractional occupancy was lower in the neuropathic pain groups compared to controls (Fig. 5d), meaning that for the neuropathic pain group, less time was spent overall in active states (Cohen’s *d* = −0.608 [95.0% CI −1.07, −0.137]; *P* = 0.0088). However, we did not find any significant differences in the switching rate between the neuropathic pain and healthy groups (two-sided permutation *t* test *p* = 0.191).

In individuals with neuropathic pain, the proportion of time spent (i.e., fractional occupancy) was higher in the SMN state and lower in the dorsal attention state compared to HCs (two-sided permutation *t* test, Cohen’s *d* > 0.519; *P* < 0.024). In addition, the interval of time between visits of the SMN state was shorter in neuropathic pain than in HCs (Cohen’s *d* = −0.508 [95.0%CI −0.95, −0.0369]; *P* = 0.028), whereas the period between visits in the dorsal attention network tended to be longer in neuropathic pain than in HCs (Fig. 5e, f; Cohen’s *d* = 0.414 [95.0% CI −0.0433, 0.872]; *P* = 0.069). The life times of the states were not significantly different between both groups, but the SMN tended to be longer in neuropathic pain. This characterized neuropathic pain as an imbalance of brain temporal dynamic between sensorimotor and frontal states (see Supplementary Fig. 2).

## Discussion

Neuropathic pain is a highly prevalent (4–8% of the population) and disabling chronic pain condition. MEG affords excellent sensitivity to probe neural function in those with neuropathic pain^14,15,44,45^, and to examine abnormal alpha neural oscillations, a potential sensitive neuromarker^3^. Here we examined abnormalities in the spatiotemporal organization of brain states in patients with neuropathic pain compared to age/sex-matched HCs using a MEG data-driven analysis based on HMMs. Brain states were defined in a full probabilistic approach using HMM, as large-scale networks becoming active with distinct spatial and spectral features that include both power and phase-coupling^32,34^ across selected brain regions of the DPC. Our study has two main findings: First, we found that resting-state brain activity in MEG can be described by 12 short-lived transient and reoccurring brain states (i.e., time-varying neural processes) that switch about every 80 ms according to the HMM parameters selected. This highlights the very fast dynamic changes of brain networks organization (in term of power and phase-coupling), similar to the rapidly changing phase-coupling activity during tasks^46^. The finding of fast timescales phenomenon of human brain activity at rest suggests this as an essential element to support efficient cognitive processes^47^.

Our second main finding is that neuropathic pain impairs the dynamic coordination of neural network activity. Compared to HCs, the neuropathic pain cohort exhibited shorter time intervals between transitions to (i.e., visits of) the SMN and their brain spent more active time in this state. Moreover, in the beta band, patients had higher power and coherence in the SMN state. Conversely, their brains showed lower coherence and spent less time in the frontal attentional state. We hypothesized that these findings illustrate an imbalance of temporal dynamics between sensorimotor and frontal microstates in neuropathic pain that may contribute to abnormal pain regulation in these patients.

In the alpha-band, neuropathic pain was associated with higher power in the ANP and stronger phase-coupling connectivity in the SN, two major brain networks involved in the conscious pain experience^4,6,48^ (see Figs. 3–5 and Supplementary Table 3).

The findings in this study suggest that the HMM method can capture the complex and rich brain dynamics underpinning neuropathic pain. Thus, HMM offers correlational markers to characterize the neuropathology of neuropathic pain as a dynamic alteration process and detect its functional consequences. This result can inform hypothesis generation for future studies that can potentially lead to developing a mechanism-based therapeutic approach. For example, identifying new brain targets that can be stimulated offers the hope of modifying or resetting abnormal activity using neuromodulation to restore effective communication between brain states.

Transient, dynamic brain events (i.e., microstates of high amplitude activity) are a fundamental mode of neural functioning^49^. MEG recordings provide a sensitive insight into micro- and macroscopic neural circuits that dynamically form and dissolve to underpin cognitive functions, by measuring the magnetic fields generated by neuronal current flow in the brain. We found that the brain network functioning in HCs and those with neuropathic pain could be temporally segregated into 12 consistent and connected patterns of brain activity during the scanning time. They correspond to commonly observed functional resting-state networks (at the spatial level within the DPC^4,6^) including the DMN, SMN, visual, frontoparietal, attentional, and ANP. The spatial maps primarily showed patterns that are in line with the previous work^32,33,50^ that examined the large-scale UK Biobank data. This provides confidence in the generalizability of the HMM in our study which had a modest sample size.

Of note is that we do not claim this 12-states solution to be more biologically relevant than 6 (see Supplementary Fig. 1), but rather that different numbers of states offer different levels of detail of brain dynamics. The visual verification confirms some consistency in the two-level analysis and was not a surprising result given the known asymmetry of the brain, with related states fused into one.

In our study, consistent with previous works^32,51^, the DMN was subdivided into two higher-order cognitive brain states, corresponding to the anterior (e.g., mPFC, ACC, dlPFC) and posterior (e.g., PCC, precuneus) subdivisions of the DMN. These two DMN states or subnetworks had particularly high power and coherence in comparison with other states and were distinguished from each other because they operated at two different frequency bands. The posterior DMN state was most pronounced within the alpha frequency band (≈12 hz), whereas the anterior DMN state was most activated in the delta/theta frequency band (≈5 hz). These two distinct functional systems and their functioning within different frequency bands may reflect different intrinsic timescales that have been proposed to specialize within the temporal domain^52^. These subsystems are composed of two central nodes within the structural core of the brain, the PCC and the medial prefrontal cortex, both highly anatomically connected. They are actively involved in the construction of self-relevant mental simulations by integrating prior experiences, the PCC maintains a sense of self-consciousness that is engaged in self-referential mental thoughts during rest^53^, and is related to positive empathy from others^54^.

We also found that the temporal dynamics of the anterior and posterior DMN were both different compared with the other states, in that they exhibited higher active time (i.e., life time), but were not revisited for longer periods and tended to be visited less frequently than other states such as the visual or SMN states. This result differs from the previous work of Vidaurre et al.^32^ who did not find significant differences in fractional occupancy of these two states compared with the other states.

The sensitivity and specificity of neurophysiological indices derived from MEG show promise to potentially assess neuropathic pain^3,13–15,44,45^. However, averaging data over the duration of test recording may result in losing major features of these transient neural signals. This limitation is not present in the neural modeling using HMM, which provides a deeper understanding of the origin of signal abnormalities within the brain. In this paper, we combined both MEG recording and HMM methods to determine the impact of neuropathic pain on dynamic neural activity and identify information about the neural impairment underlying neuropathic pain.

Abnormalities in neural oscillations are an indication of pathology—putative oscillopathies—and several atypical oscillatory signatures of neuropathic pain have been reported. Previous studies have demonstrated that there is pathological slowing and increased power of the peak alpha frequency in chronic neuropathic pain, associated with higher trait pain intensity in nodes of the SN (TPJ) and the ANP (posterior insula)^3,14,15,55^. Our findings are consistent with these previous findings and suggest that the largest spectral power difference in alpha amplitude between people with neuropathic pain and HCs occurs during the active state composed of nodes of the right ANP, including higher power in regions particularly involved in the discriminative somatosensory aspects of pain (e.g., TPJ, the posterior insula, S1, and M1), and lower power in regions involved in the modulation of nociceptive information (e.g., the DLPFC, mPFC, and anterior insula; see Fig. 3 and Supplementary Table 3)^4,48,56^.

Interestingly, alpha activity is thought to reflect active control of information flow in the working brain through functional inhibition of task-irrelevant regions^57^. It has also been proposed to represent different brain attentive states, which have a bias either toward external or internal processing^58^. Therefore, the observed changes in alpha power when the ANP state is active could indicate abnormalities of neural resources allocation in the resting brain of people with neuropathic pain, which change the system toward processing information inwardly, rather than controlling sensory gating (i.e., external processing through sensory channels).

In the same vein, we found atypical functional coherence over the brain microstates in neuropathic pain. Post hoc analyses showed that the most affected connections were in the salience state in alpha (increased) and in the dorsal attention state in delta/theta frequency band (decreased), between regions of polymodal cortices compared to HCs (see Fig. 4 and Supplementary Table 3). These well-known foci of attentional processes are greatly altered in neuropathic pain^15,45,59^. A recent electroencephalography study has also shown abnormalities in brain microstates related to attentional function in patients with chronic pain^60^. Brain state changes in the oscillatory communication between regions may represent a shift in the micro-temporal brain dynamics. Therefore, neuropathic pain may impact the brain's capacity to maintain proper synchronization of neural assemblies related to attentional load at rest. It has been suggested that this pronounced dysregulated coupling could be an indicator of neuropathic pain^3^. This also demonstrates the utility of a data-driven method to determine which connections are most affected and may inform hypotheses of future studies targeting dysrhythmic activity.

We have recently reported extensive reorganization of the sensorimotor subsystem, such as hyper-connectivity of the SMN, and altered hub topology in chronic pain^6^. The abnormal SMN beta power activity could be due to cortical plasticity after a lengthy period of nociceptive input^61^, or it could also serve as a compensatory mechanism to maintain sensory perception. The beta band plays a crucial role in the establishment of canonical resting-state networks^62^, and therefore can be seen to be important for information processing within and across cortical circuits^63^. Although the neurophysiological meaning behind beta band discrepancies is unclear, it illustrates that neuropathic pain has an impact on the overall dynamic orchestration of neural activity^63^.

We found that the subjective experience of chronic neuropathic pain results from pathological brain states interactions, but also from their temporal dynamic organization. The heterogeneity of clinical sequelae of neuropathic pain is thought to be driven by diffuse and widespread structural and functional damages, particularly in the SMN and descending anti-nociceptive pathway, affecting communication between distal, functionally specific regions involved in the control and modulation of pain^6,23^. Therefore, it has been proposed that chronic pain results from an imbalance between pain input evoking the somatosensory cortex and brain regions involved in pain control (i.e., inhibition through subgenual anterior cingulate cortex)^23^. A balance between areas involved in pain input and pain suppression requires temporal organization, which can be characterized by the state active duration and the interval of time between visits to brain microstates.

We observed that in individuals with neuropathic pain compared with HCs, the proportion of time spent and the frequency of visits to the sensorimotor state was higher, whereas these temporal aspects was lower in the dorsal attention state (e.g., sgACC mediated pain inhibition), illustrating a pathological imbalance of the brain temporal dynamic (Fig. 5d–f; Supplementary Fig. 2). This imbalance was also noticeable in the beta spectral power (with higher and lower power, respectively in somatosensory nodes and frontal nodes). All of these changes contribute to impaired routing of information flow between brain areas of the DPC^4^ involved in pain somatosensory-motor processing and pain suppression^12^ at rest in people with neuropathic pain, who feel pain in the absence of stimulation. The SMN is widely used as a target in neuromodulation through brain stimulation^64^, and one possible extrapolation of our findings to explain its analgesic effect can be that the stimulation could restore a proper balance of activity between brain states (i.e., between pro- and anti-nociceptive subsystems).

The HMM methodological approach overcomes the limitations of clustering and sliding-window approaches regarding the selection of the optimal window size and clustering dimensionality^65^. This work focused on lower frequency bands (1–45 Hz), due to the relatively low signal-to-noise ratio in higher-frequency bands (see methodological considerations in the methods section and here^32^), but we would expect that there are state-specific differences in gamma, because of its role in cognitive processes related-to-pain. Comparing our results with Vidaurre et al.^32^, we observed a number of similarities and only very few exceptions as described above. These mismatches can be explained not only by the different sample sizes, but also by the different ages of the sample. The brain parcellation was also different because we based our analysis on nodes of the DPC. The HMM can capture the heterogeneity of the time-dynamic variation of the neuronal activity by considering in the same framework of analysis the variability between individuals in a population. We also note that we examined only relative differences between the states (i.e., relative to other states or other subjects). This is because the absolute value of the temporal features must be interpreted with caution given the state exclusivity assumption of the HMM. Thus, this is not necessarily a physiologically meaningful feature of the brain because the state temporal information depends on the choices of prior parameters such as the distribution of the transition probability matrix.

Neuropathic pain continues to be a challenge to treat clinically. In conclusion, characterizing neuropathic pain based on electrophysiological measures may contribute to better subtyping, better diagnostic measures, and better treatments for patients suffering from this type of chronic pain. The present results demonstrate that the HMM approach for MEG data can capture the complex and rich temporal dynamics of brain microstates underpinning neuropathic pain. We propose that the subjective experience of chronic neuropathic pain results from a pathological imbalance in the dynamic spatiotemporal organization of brain states. This can have implications for the development of a mechanism-based therapeutic approach by identifying specific brain targets to stimulate using neuromodulation to modify abnormal activity and restore effective neuronal synchrony between brain states.

## Methods

### Participants

This study included 40 patients diagnosed with neuropathic pain (20 males (mean age in years ± SD = 41.4 ± 8.3) and 20 females (mean age in years ± SD = 42.7 ± 9.3); range [24–63]) and 40 age- and sex-matched HCs (20 males (mean age = 40.9 ± 9.6), 20 females (mean age = 41.6 ± 8.1); range [24–59]). All participants provided informed, written consent to procedures approved by the Research Ethics Boards of the University Health Network, and St. Michael’s Hospital. Participants were asked to refrain from caffeine and alcohol on the day of testing. The inclusion criteria for the patients included (1) chronic pain for a duration of 6 months or longer (mean pain duration in years ± (SD) in females = 12.7 ± 8.3; and males = 12.8 ± 8.0), (2) moderate-to-severe average pain intensity over a month (i.e., trait pain) rated on a scale from 0 (no pain) to 10 (worst pain imaginable), and (3) presence of clinical symptoms typical of neuropathic pain (diagnosed based on medical history; details are provided in Supplementary Note 1).

### Preprocessing of MEG data

We acquired, prior to the MRI scan, a 5-minute resting-state MEG scan with a 306 channel Elekta Neuromag TRIUX system, with a sampling rate of 1000 Hz and a DC bandpass of 330 Hz. The patients selected for analysis did not report any movement or muscle disorders that may have created artifacts. Fiducial reference points were marked at the nasion and bilateral pre-auricular positions for motion correction and registration to the MRI anatomical scan^66^. MRI scanning was done with a 3 T device fitted with an eight-channel phase array head coil (GE Medical Systems) to acquire a high-resolution T1-weighted anatomical scan (3D IR-FSPGR sequence; 180 axial slices; TR, 7.8 ms; TE, 3 ms; flip angle, 15°; 256 × 256 matrix; voxel size, 1 mm^3^). Participants were scanned sitting in an upright position in the MEG with their eyes open and fixated on a cross on a screen in front of them inside a dark room. Instructions were to stay still, relax, avoid structured thinking and to let their mind wander. The position of the participant’s head was monitored continuously through 5 head position indicator coils. We used the spatiotemporal signal space separation algorithm^67^, implemented in the MaxFilter program of the MEG system, for artifact removal and head movement correction.

This analysis depends on several toolboxes and software packages (freely available). The preprocessing and source-space parcellation analyses are performed using the Fieldtrip, SPM, FSL(v5.0), and some part of the OHBA Software Library (OSL; https://ohba-analysis.github.io/osl-docs/), using the most adapted tool for each step of the preprocessing and analysis.

MEG resting-state data were preprocessed using previous published methods^14,15^. We used the Fieldtrip toolbox (http://www.fieldtriptoolbox.org/) run on MATLAB software and data were downsampled to 300 Hz, bandpass filtered at 1 to 150 Hz, a notch filter was applied at 60 and 120 Hz. The first and last 10 seconds of the recorded data were removed, leaving 280 s of resting-state data for each participant. Independent component analysis was used (runica function) to remove artifacts associated with cardiac artifacts, eye blinks, breathing and muscle activity, as identified by visual inspection. For each individual, fiducial points (i.e., nasion and bilateral pre-auricular) were identified on the anatomical T1 image, and these were used to co-register the participant’s resting-state MEG data to their own MRI anatomical image. After co-registration, each individual preprocessed data is warped into a template brain. The anatomical image was then segmented using statistical parametric mapping, resulting in a geometrical representation of the brain which was then used in a realistically shaped single-shell forward model^68^. We used a linearly constrained minimum variance beamformer^69^ to extract a continuous time series for 36 nodes of the DPC^4,6,14,15,39,45^ covering the entire cortex (cortical and subcortical regions of interest used as virtual sensors for the atlas-guided beamforming are derived from the MNI coordinates listed in Supplementary Table 1). The DPC concept is a well-accepted description of the system of brain regions that shape the overall experience of pain including the SN, DMN, and ascending and descending nociceptive pathways^4,70^.

Bad segments were removed manually and a symmetric multivariate leakage correction for MEG connectomes was applied using the technique described in ref. ^71^.

### The time-delay embedded HMM

The HMM statistical approach is composed of two components: The first is a Markov chain, a sequence of short-time stationary events characterizing the evolution of neuronal activity. The second component of the model, a set of output distributions, hides this sequence of states from the observer, which controls how the sequence of states is converted into a sequence of MEG observations.

In this study, we used a Time Delay Embedded HMM^32^ to characterize spectrally resolved networks characterized by power-spectral densities and phase-locking. It infers a multivariate Gaussian distribution describing a delay-embedding of the source time-courses and is appropriate for application to large-scale brain networks inferred from parcellated source-space MEG data. HMM is a probabilistic model assuming that brain time series is composed of a sequence of states such that, at each time point, only one state is active. Importantly, the probability of a state being active at time point *t* is modeled to be dependent on which state was active at time point *t*−1 (i.e., it is order-one Markovian).

The HMM is inferred using the HMM-MAR toolbox https://github.com/OHBA-analysis/HMM-MAR, through the hmmmar.m function (details about the options can be found here https://github.com/OHBA-analysis/HMM-MAR/wiki/User-Guide#-hmm-marmodel-estimation)

### Data processing

The HMM state distribution was applied to the concatenated data of all subjects to obtain a group estimation of the states (i.e., HCs and neuropathic pain), but importantly the individual information of a state time course is still available.

Since the beamforming process is done for each subject independently, the sign of source-localized MEG data is arbitrary and can be inconsistent across subjects. This can lead to the suppression of connectivity between any pair of regions at the group level. Therefore, this is crucial to resolve the ambiguity of the source polarity, and for that, we applied the sign-flipping algorithm described in^32^.

The source-reconstructed time courses for each parcel were time-delay embedded using L lags. Here, we covered a window of 15 time points around the time point of interest (*L* = 15), with values between −7 and 7. Then, the HMM is run on a principal component analysis aiming to explain the highest possible amount of variance in the time series. In general, fewer components will bias the HMM towards lower frequencies because lower frequencies tend to explain more variance in the data. We used twice PCs as the number of regions (i.e., 72 PCs). This explains on average 68% of the variance.

### Observation model

We used the same option settings as in^32^, and the observation model for each of the 12-states is defined as a multivariate normal distribution. The oscillatory signals are emphasized within the MEG source-space data, and only the covariance matrix within each state is modeled (i.e., zero mean). The brain activity is modeled over a certain time window, thus the observation model corresponded to the autocovariance matrix across brain regions within such window. Here we used a window of 50 ms and 36 × 2 PCs to be better able to capture higher-frequency differences.

### Stochastic inference

The HMM analyses can be very computationally intensive, thus we adapted the stochastic inference batch settings and used Linux workstations with an Intel Xeon E5 CPU clocked at 1.90 GHz running in parallelization and 16 Gb of RAM.

### Run-to-run variability

To test the stability of the HMM results across several runs of the inference, the HMM is run multiple times and the result of each iteration is compared using the free-energy value. The model with the lowest free-energy can be taken as the one which best explains the data without becoming too complex. We repeated the HMM inference ten times and the analysis proceeded with the iteration with the lowest free energy.

### Extracting spectral information

Once the HMM model is trained and the state time courses obtained, we estimated the spectral content of the model, i.e., power-spectral density and coherence. To do that, we used a nonparametric estimation, using a state-wise multitaper approach, which provided power and spectral coherence for each frequency bin between 1 and 45 Hz (introduced in ref. ^34^).

The spectral information computed contains (states by frequency bins by channels by channels) a lot of values. This is an overwhelming amount of information for ease of interpretation. For this reason, power and spectral coherence information for each state were factorized into different frequency bands. We performed this frequency decomposition in a data-driven way as in Vidaurre et al.^32^, we applied the factorization to the spectral estimation data of each subject. To do that, we used non-negative matrix factorization as the decomposing method and asked for four modes or frequency bands (instead of having for instance 300 frequency bins). Note that this estimation depends on an optimization process with a random initialization; therefore, we inspected the spectral profiles visually and rerun if the frequency modes were too unclear. The non-negative matrix factorization was run several times, and the solution with the most clearly unimodal modes (i.e., with just one peak per mode) was the chosen one. Four bands solution was the one chosen, instead of three or five, because the result gave stable decomposition matching reasonably with the classical frequency range used in the literature (i.e., delta/theta, alpha, beta, gamma).

### HMM global temporal statistics

The HMM inference provides the state time courses indicating the probability of each state to be active at each time point, and the description of the probability distribution of each state. We computed the following temporal metrics to interrogate the results and characterize the dynamic properties of the brain states: (1) the transition probabilities from any state to any other state, without considering the persistence probabilities (i.e., the probability to remain in the same state); (2) the state time fractional occupancies, which refers to how much time each subject spends in each state (i.e., the average state probability across time, per subject); (3) the state switching rates for each subject, and can be understood as a measure of stability per subject; (4) the state lifetimes, which reflects the temporal stability of the states; (5) the state interval times containing the number of time points between visits.

### Statistical analysis

We performed nonparametric statistical testing to investigate which spectral information (functional coherence or power) was either significantly stronger for any given state with respect to the other states, or significantly different in the neuropathic pain group compared with HCs. We calculated the spectral information for each subject separately and then used this between-subject variability to run a standard permutation testing analysis.

At each permutation, we shuffled the target power or functional connection value across states. By running 5000 permutations, we created (for each power and functional connection value) a null distribution of differences between each state’s value and the mean value of the other states, which we then used to produce a *P* value per activation value and functional connection, corrected for multiple comparisons.

To examine group differences in the spectral information we compared the entire group of patients with neuropathic pain with the group of HCs using two-sided permutation *t* test, 5000 bootstrap samples were taken. To do that, we shuffled (5000 times) the group belonging to the subjects and compared the original difference between groups to the distribution of differences after permutation. Effect sizes for the peak alpha were computed with the Cohen *d*, and the confidence intervals were bias-correlated and accelerated. In addition, to avoid false-positive results due to pure chance, when appropriate, we corrected the *P* value for multiple comparison according to the number of states x frequency bands using false discovery rate (FDR) with the Benjamin–Hochberg method at FDR 0.05.

Finally, we used permutation analysis using Network-based statistic^41,42^ (NBS toolbox; https://www.nitrc.org/projects/nbs) to identify local nodal changes of power and coherence between neuropathic pain and HCs in brain states and frequency bands associated with neuropathic pain abnormalities.

Permutation analysis using a network-based statistic approach is increasingly being used to identify differences in large-scale brain connectivity networks. The NBS is a nonparametric statistical method to deal with the multiple comparisons problem when there are a large number of connections and is well-suited for identifying connections that may be associated with a between-group difference (i.e., patient vs control) in clinical studies^42^. The method is used to control the family-wise error rate (FWER), and the NBS exploits the extent to which the connections comprising the contrast are interconnected to offer a substantial gain in power. FWER-corrected p-values are calculated for each set of interconnected regions of interest using permutation testing. Moreover, NBS was used at the nodal level because of the expected nonnormal distribution of differences in brain microstates measures.

Statistical analyses were performed using MATLAB software (R2019b, Math-Works, Naticks), the estimation statistics^72^ package under Python (https://github.com/ACCLAB/DABEST-python; for more details also see https://www.estimationstats.com) and GraphPad Prism (version 7.03; www.graphpad.com).

### Reporting summary

Further information on research design is available in the Nature Research Reporting Summary linked to this article.

## Supplementary information

Supplemental Information

Description of Additional Supplementary Files

Supplementary Data 1

Reporting Summary

## Data Availability

The MEG data that support the findings of this study are available upon reasonable request from the corresponding author (Dr. Karen Davis). The data are not publicly available due to third-party restrictions and patient privacy issues of the institution. Source data underlying figures are provided in Supplementary Data 1.

## Code availability

All analyses were done using freely available tools in MATLAB. The code used to conduct the analysis in this paper can be found at: https://github.com/OHBA-analysis/HMM-MAR Information can also be found here: OHBA Software Library (OSL; https://ohba-analysis.github.io/osl-docs/). Furthermore, we used the scripts previously published by Vidaurre et al.^32^ using the OHBA toolbox, which is freely available. The script containing the entire pipeline is available from the original work of Vidaurre et al.^32^ (https://github.com/OHBA-analysis/HMM-MAR/blob/master/examples/NatComms2018_fullpipeline.m) which can be used to reproduce the methodological approach.
