# Supplementary material for: A Hidden Markov Model reveals magnetoencephalography spectral frequency-specific abnormalities of brain state power and phase-coupling in neuropathic pain
Source: Commun Biol. 2022 Sep 21;5:1000. doi: 10.1038/s42003-022-03967-9 (PMC9492713; doi:10.1038/s42003-022-03967-9)
Supplement: Supplementary file 1 — Supplemental Information [file 42003_2022_3967_MOESM1_ESM.pdf]

A Hidden Markov Model reveals magnetoencephalography spectral frequency-specific abnormalities of brain state power and phase-coupling in neuropathic pain

Camille Fauchon[1], Junseok A. Kim[1,2], Rima El-Sayed[1,2], Natalie R. Osborne[1,2], Anton Rogachov[1,2], Joshua C. Cheng[1,2], Kasey S. Hemington[1,2], Rachael L. Bosma[1], Benjamin T. Dunkley [3,4,5], Jiwon Oh[6], Anuj Bhatia[1,7], Robert D. Inman[2,8], and *Karen Deborah Davis[1,2,9]

[1]Division of Brain, Imaging, and Behaviour, Krembil Brain Institute, University Health Network, Toronto, ON, Canada M5T 2S8

[2] Institute of Medical Science, University of Toronto, Toronto, ON, Canada M5S 1A8

[3] Neurosciences & Mental Health Program, The Hospital for Sick Children Research Institute, Toronto, ON, Canada M5G 0A4

[4] Diagnostic Imaging, The Hospital for Sick Children, Toronto, ON, Canada M5G 0A4

[5] Department of Medical Imaging, University of Toronto, Toronto, ON, Canada M5T 1W7

[6] Div of Neurology, Dept of Medicine, St. Michael's Hospital, Toronto, ON, Canada M5B 1W8

[7] Department of Anesthesia and Pain Medicine, Toronto Western Hospital, and University of Toronto, Toronto, ON, Canada M5T 2S8

[8] Division of Immunology, University of Toronto, Toronto, ON, Canada M5S 1A8

[9] Department of Surgery, University of Toronto, Toronto, ON, Canada M5T 1P5

*Corresponding author:

Karen D. Davis, PhD, FCAHS, FRSC

Division of Brain, Imaging, and Behaviour, Krembil Brain Institute, University Health Network

399 Bathurst St, Room MP12-306; Toronto, ON M5T 2S8, Canada

Tel: (416) 603-5662; E-mail address: karen.davis@uhnresearch.ca

Supplementary Note 1

Participants –additional information

The neuropathic pain group consisted of 11 patients with ankylosing spondylitis (5 males), 10 with multiple sclerosis (6 males), 10 with carpel tunnel syndrome (4 males), and 9 with other neuropathic etiologies (5 males). Although ankylosing spondylitis was classically considered to involve inflammatory pain, recent studies have demonstrated that there is a much higher incidence of neuropathic pain than previously thought[1]. Many patients with ankylosing spondylitis (and other inflammatory and degenerative musculoskeletal conditions including osteoarthritis) have a strong component of neuropathic pain, and this is often associated with more severe brain abnormalities [2, 3]. Thus, recent studies have considered ankylosing spondylitis a mixed neuropathic pain-inflammatory condition[4].

Symptoms of neuropathic pain included clinical features such allodynia, hyperpathia, continuous pain and/or presence of spinal or brain lesions. Consistent with our previous studies, we considered patients with clinical evidences and also a score higher than 13 on the painDETECT questionnaire[5] to likely have a component of neuropathic pain[1, 2, 3, 6, 7].The painDETECT scores range from 0-38 and includes an assessment of qualities of pain that are typically associated with neuropathic pain (e.g., burning, tingling, electric shocks).

Further inclusion criteria for all participants were: (1) 18-65 years old, (2) no prior diagnosis of neurological, psychiatric, or metabolic conditions (e.g., diabetes, and for healthy controls no previous history of chronic pain or current experience of pain on a regular basis), (3) no major surgery in the past 2 years or taking medications on a regular basis (except for the chronic pain-related treatments) prior to study participation, and (4) no standard contraindications to MRI or MEG.

The participants with neuropathic pain had undergone a variety of treatments including use of medications such as tricyclic antidepressants (e.g., teva-Amitriptyline), selective serotonin

reuptake inhibitors (SSRIs), serotonin and norepinephrine reuptake inhibitors (SNRI) antidepressants (e.g., duloxetine, venlafaxine), cannabinoids, antiepileptics (e.g., pregabalin, gabapentin), benzodiazepines, opioids (e.g., oxycodone), biologics (e.g., tumor necrosis factor (TNF)-alpha inhibitors such as Enbrel, Humira, Remicade, and Simponi), grade 2 analgesic drugs including Tylenol and non-steroidal anti-inflammatory drugs (i.e., Voltaren, Naprosyn, and Celebrex) and nonpharmacological treatments (e.g., acupuncture, physical therapy, TENS). It was not possible to quantify the medication(s) for the purposes of the current study because each participant used various medications that varied over time. The Hospital Anxiety and Depression Scale (HADS)[8] was self-administered by the participants to assess nonphysical symptoms of anxiety and depression. Scores > 8 were considered clinically significant, but it is possible that subclinical levels could have been present (males and females with neuropathic pain: HADS depression (/21) = 8 ± 5; and HADS anxiety (/21) females with neuropathic pain = 7 ± 4 and males with neuropathic pain = 8 ± 4).

Few studies have shown that sex hormone level relative to the menstrual cycle phase influence the frequency of alpha oscillation[9] and brain activity in general[10]. The females' hormonal profile was very diversified (i.e., various phase of menstrual cycle, and included eumenorrheic and postmenopausal women) in both healthy females and those with neuropathic pain. Therefore, although hormonal factors could contribute to the individual variability within the female subgroup, it is unlikely that this impacted the results.

For spatial, spectral, or temporal measures that had an effect of neuropathic pain (i.e., group differences between neuropathic pain and matched-healthy controls), we used Pearson correlation to assess the relationship between individual variability in these variables and neuropathic pain clinical pain measure (painDetect scores and ratings of average pain experienced in the last 4 weeks – i.e., trait pain). We did not find any significant correlations after correction of P-values for multiple comparison. This may in part be due to our modest sample size and homogeneity of our patient group.

Supplementary Table 1

We used a linearly constrained minimum variance beamformer[11] to extract a continuous time series for 36 nodes of the dynamic pain connectome. Beamforming is a spatial filtering technique that is used in our approach to isolate the signal of interest corresponding to a voxel at the center of mass of each regions of interest (ROI) while optimally suppressing the signals from other sources[12, 13]. To do this, a weighting vector was calculated for each source location in the brain and applied to the physical sensor's time course. The resultant time series were summated and give a reconstructed signal for the specified source location over time[11].

We selected 36 region of interests within the dynamic pain connectome (DPC) based on previously defined coordinates[2, 3, 14, 15, 16] and these were used as "virtual sensors" for the atlas-guided beamforming. The ROIs were visually confirmed on a standard MNI152 anatomical template (coordinates x, y z), and included the following areas.

Index	*Region of interest (abbreviation)*	*MNI coordinates (x,y,z)*
1	Left Thalamus	(-12,-18,8)
2	Right Thalamus	(12,-18,8)
3	Left primary somatosensory cortex (S1)	(-34,-30,54)
4	Right primary somatosensory cortex (S1)	(34,-28,54)
5	Left secondary somatosensory cortex (S2)	(-60,-30,20)
6	Right secondary somatosensory cortex (S2)	(60,-22,18)
7	Left posterior insula (pINS)	(-34,-20,18)
8	Right posterior insula (pINS)	(34,-20,18)
9	Left temporo-parietal junction (TPJ)	(-50,-42,28)
10	Right temporo-parietal junction (TPJ)	(50,-32,28)
11	Left anterior insula (aINS)	(-34,18,4)
12	Right anterior insula (aINS)	(34,18,4)
13	Mid-cingulate cortex (MCC)	(2,12,34)
14	Left dorsolateral predrontal cortex (dlPFC)	(-38,40,28)
15	Right dorsolateral predrontal cortex (dlPFC)	(34,46,22)
16	Posterior cingulate cortex (PCC)	(-2,-46,28)
17	Medial prefrontal cortex (mPFC)	(-2,50,2)
18	Subgenual anterior cingulate cortex (sgACC)	(4,26,-8)
19	Left Amygdala	(-24,-1,-17)
20	Right Amygdala	(26,1,-18)
21	Left primary motor cortex (M1)	(-40,-6,51)
22	Right primary motor cortex (M1)	(40,-8,52)
23	Left sensorimotor area (SMA)	(-6,5,61)
24	Right sensorimotor area (SMA)	(8,0,62)
25	Left Caudate	(-12,11,9)
26	Right Caudate	(14,12,9)
27	Left Putamen	(-25,4,2)
28	Right Putamen	(27,5,2)
29	Left Pallidum	(-19,0,0)
30	Right Pallidum	(20,0,0)
31	Precuneus	(2,-61,48)
32	Dorso-medial prefrontal cortex (dmPFC)	(-13,52,23)
33	Left occipital cortex (Occi)	(-14,-94,24)
34	Right occipital cortex (Occi)	(10,-94,24)
35	Left medial temporal lobe (MTL)	(-62,-22,8)
36	Right medial temporal lobe (MTL)	(58,-22,8)

Supplementary Table 2

Nodes showing significant power in each state in both healthy controls and neuropathic pain groups are listed below. Each state is characterized by specific significant power in several ROI relatives to the average power across states (non-parametric statistical testing on the between-subject variability).

MEG brain microstates	Nodes' positive power	Nodes' negative power
Posterior default mode network (DMN)	*L_S1; R_S1; PCC Precuneus; L_occi; R_occi*	*L_aINS; L_DLPFC; R_DLPFC; mPFC; sgACC; L_ Amygdala; R_ Amygdala; R_SMA; L_Caudate; L_MTL*
Anterior default mode network (DMN)	*L_aINS; R_aINS MCC; L_dlPFC; R_dlPFC; mPFC; sgACC; L_SMA; R_SMA; L_Caudate; R_Caudate; dmPFC*	*L_S1; R_S1; L_S2; R_S2; L_pINS; R_pINS; L_TPJ; R_TPJ; L_M1; R_M1; R_occi; R_MTL*
Dorsal attention	*L_aINS; R_aINS; L_DLPFC; R_DLPFC; L_ Amygdala; L_caudate; L_putamen; R_putamen; L_Pallidum; L_MTL*	*R_Thalamus; R_S1; R_S2; R_pINS; R_TPJ; MCC; PCC; mPFC; R_M1; L_M1; L_SMA; R_SMA; Precuneus; L_occi; R_occi; R_MTL*
Fronto-parietal	*R_S1; mPFC; sgACC L_M1; R_M1; R_SMA; Precuneus; L_occi*	*L_S2; L_pINS; R_aINS; R_ Amygdala; R_caudate; R_Putamen; R_Pallidum; L_MTL*
Motor-control	*R_S1; MCC; mPFC; Amygdala_L; Precuneus; L_M1; R_M1; L_SMA; SMA; L_Caudate*	*L_Thalamus; L_S1; L_S2; L_pINS; L_TPJ; R_aINS; R_Putamen; R_Pallidum; L_MTL*
Salience	*MCC; dmPFC; R_aINS; L_aINS; R_pINS; R_S2; R_TPJ; R_ Amygdala; R_MTL*	*SgACC; R_caudate; L_caudate; MCC; PCC; R_S1; Precuneus; R_SMA; L_SMA*
Left ascending nociceptive pathway (ANP)	*L_Thalamus; L_S2; L_pINS; L_aINS; MCC; sgACC; L_Amygdala; R_SMA; L_Putamen; L_Pallidum; L_MTL*	*R_Thalamus; L_S1; R_S1; R_pINS; R_TPJ; PCC; R_Pallidum; Precuneus; dmPFC; L_occi; R_occi*
Right ascending nociceptive pathway (ANP)	*R_S1; R_S2; R_TPJ R_aINS; R_Amygdala R_M1; R_Caudate; R_MTL; R_Putamen; R_Pallidum*	*L_Thalamus; L_S2; L_pINS; MCC; L_DLPFC; mPFC; L_SMA; Precuneus, dmPFC; L_occi; R_occi; L_MTL*
Right Operculo-insular	*R_Thalamus; R_S2; R_pINS; R_TPJ; R_SMA; R_MTL*	*R_S1; L_S1; R_M1; L_M1; L_TPJ; L_S2; L_pINS; L_Thalamus; R_aINS; R_DLPFC; mPFC*

Left Operculo-insular	*L_Thalamus; L_S2; L_S1 L_pINS; L_TPJ; L_M1; L_MTL*	*Precuneus; L_SMA; MCC; sgACC; L_aINS; L_Amygdala; L_Caudate; L_Putamen; L_Pallidum*
Sensorimotor (SMN)	*L_S1; R_S1; L_M1; R_M1; L_SMA; MCC; R_SMA; PCC; Precuneus; L_occi*	*L_S2; R_S2; L_aINS; R_aINS; L_Amygdala; R_ Amygdala; R_SMA; R_Pallidum; R_Caudate; R_Putamen; L_MTL*
Visual	*R_occi; L_occi; L_MTL*	*R_DLPFC; L_DLPFC; L_Caudate; L_aINS L_ Amygdala; L_TPJ; L_Pallidum; L_Putamen*

Supplementary Table 3

Nodes showing significant power, functional coherence, and temporal abnormalities (permutation tests, network-based and estimation statistics (Cohen's d), $P_{corrected} < 0.05$) within or between states in neuropathic pain (NP) compared with healthy control (HC) group across three frequency bands (i.e., delta/theta, alpha, beta bands)

Variables	Frequency band	MEG brain microstates	Higher in NP vs. HC	Lower in NP vs. HC
**Power**	Alpha	Right ascending nociceptive pathway (ANP)	thalamus, S1, pINS, TPJ, M1	DLPFC, mPFC, left aINS
	Beta	Sensorimotor (SMN)	S1, TPJ	DLPFC, mPFC, dmPFC, sgACC, MCC, SMA
**Functional coherence**	Delta/theta	Dorsal attention		sgACC, MCC; right: aINS, dlPFC, pallidum, putamen
	Alpha	Salience (SN)	left: S1, pINS, aINS, thalamus, caudate, mPFC, MCC, sgACC; right: DLPFC, M1, MTL, pINS, S1, PCC, Precuneus, occi	
	Beta	Sensorimotor (SMN)	dmPFC, mPFC, sgACC, MTL, pallidum, putamen, caudate Left: occi, TPJ, S1, M1, DLPFC, right: amygdala, S2	
**States temporal dynamic**	NA	Global maximum fractional occupancy		less time was spent in global in active states in individuals with NP
		Sensorimotor (SMN)	Fractional occupancy (FO)	Time interval
		Dorsal attention		Fractional occupancy (FO)

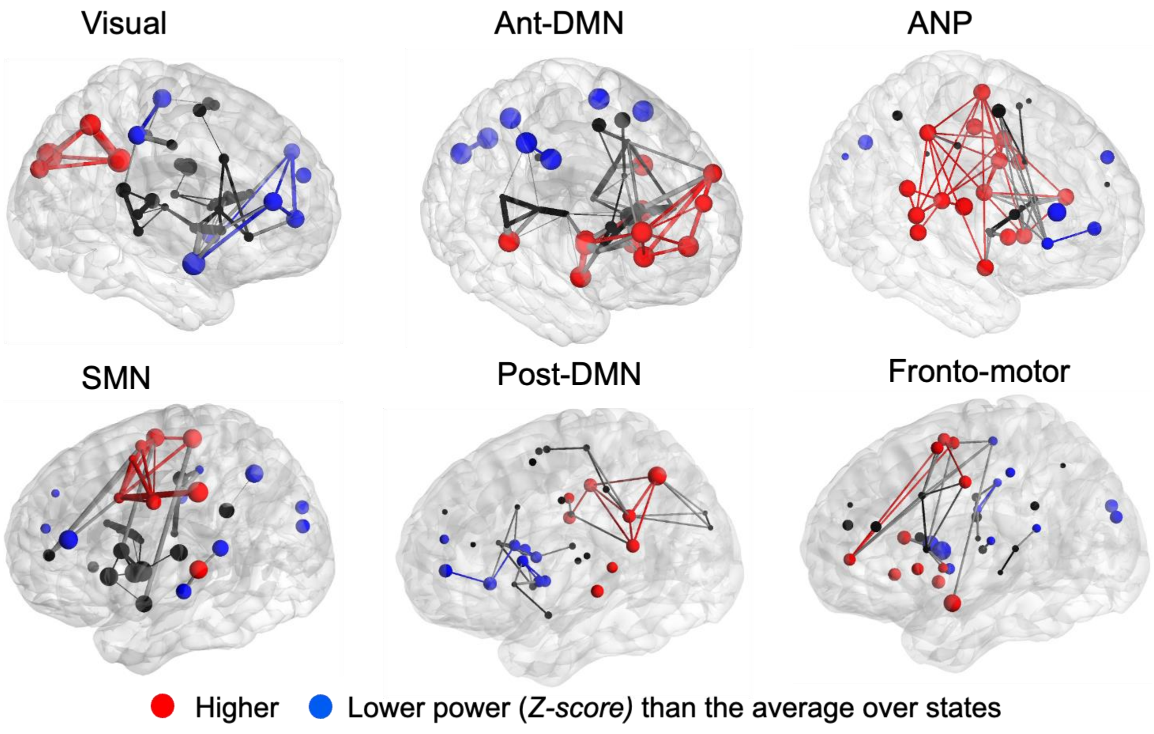

Supplementary Figure 1.

Spatial power and coherence maps for an HMM run with 6 states solution.
Some states from the original 12 state analysis are now combined into fewer states. Node spectral power is relative to the temporal average, and node size is in relation to the mean power (Z-score) across states (blue and red colours reflect power that is lower or higher than the average over states, respectively). Edges between nodes show functional connectivity, and only significant and high-valued connections are shown. DMN: default mode network; ANP: ascending nociceptive pathway; SMN: sensorimotor network.

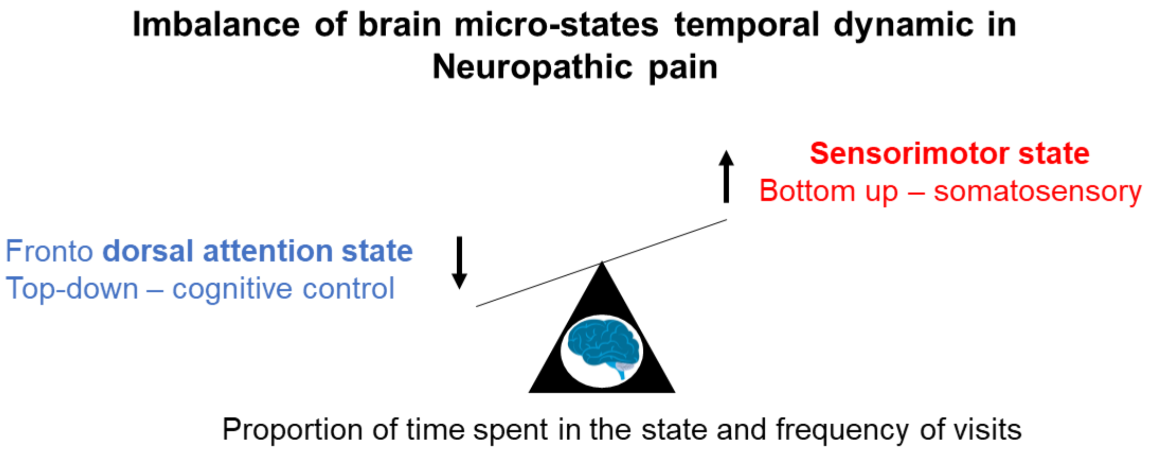

Supplementary Figure 2.

Neuropathic pain as an imbalance of brain temporal dynamic between sensorimotor and frontal states. In the neuropathic pain group, the proportion of time spent (fractional occupancy) and the frequency of visits (interval of time between visits) of the sensorimotor (SMN) state were higher, whereas these temporal aspects were lower in the dorsal attention state compared with healthy controls group, illustrating a pathological imbalance of the brain state temporal dynamic.

Supplementary References

1. Wu Q, Inman RD, Davis KD. Neuropathic Pain in Ankylosing Spondylitis: A Psychophysics and Brain Imaging Study. *Arthritis & Rheumatism* **65**, 1494-1503 (2013).

2. Kisler LB, *et al.* Abnormal alpha band power in the dynamic pain connectome is a marker of chronic pain with a neuropathic component. *Neuroimage Clin* **26**, 102241-102241 (2020).

3. Kim JA, *et al.* Neuropathic pain and pain interference are linked to alpha-band slowing and reduced beta-band magnetoencephalography activity within the dynamic pain connectome in patients with multiple sclerosis. *Pain* **160**, 187-197 (2019).

4. Bidad K, Gracey E, Hemington KS, Mapplebeck JCS, Davis KD, Inman RD. Pain in ankylosing spondylitis: a neuro-immune collaboration. *Nature Reviews Rheumatology* **13**, 410-420 (2017).

5. Freynhagen R, Baron R, Gockel U, Tölle TR. painDETECT: a new screening questionnaire to identify neuropathic components in patients with back pain. *Current Medical Research and Opinion* **22**, 1911-1920 (2006).

6. Bosma RL, *et al.* Dynamic pain connectome functional connectivity and oscillations reflect multiple sclerosis pain. *PAIN* **159**, 2267-2276 (2018).

7. Cheng JC, *et al.* Multivariate machine learning distinguishes cross-network dynamic functional connectivity patterns in state and trait neuropathic pain. *PAIN* **159**, 1764-1776 (2018).

8. Zigmond AS, Snaith RP. The hospital anxiety and depression scale. *Acta Psychiatr Scand* **67**, 361-370 (1983).

9. Brötzner CP, Klimesch W, Doppelmayr M, Zauner A, Kerschbaum HH. Resting state alpha frequency is associated with menstrual cycle phase, estradiol and use of oral contraceptives. *Brain Research* **1577**, 36-44 (2014).

10. Pletzer B, Harris T-A, Scheuringer A, Hidalgo-Lopez E. The cycling brain: menstrual cycle related fluctuations in hippocampal and fronto-striatal activation and connectivity during cognitive tasks. *Neuropsychopharmacology* **44**, 1867-1875 (2019).

11. Van Veen BD, van Drongelen W, Yuchtman M, Suzuki A. Localization of brain electrical activity via linearly constrained minimum variance spatial filtering. *IEEE Trans Biomed Eng* **44**, 867-880 (1997).

12. Sekihara K, Nagarajan S, Poeppel D, Miyashita Y. Reconstructing spatio-temporal activities of neural sources from magnetoencephalographic data using a vector beamformer. *Proceedings - ICASSP, IEEE International Conference on Acoustics, Speech and Signal Processing* **3**, 2021-2024 (2001).

13. Hillebrand A, Barnes GR, Bosboom JL, Berendse HW, Stam CJ. Frequency-dependent functional connectivity within resting-state networks: an atlas-based MEG beamformer solution. *Neuroimage* **59**, 3909-3921 (2012).

14. Kucyi A, Salomons TV, Davis KD. Mind wandering away from pain dynamically engages antinociceptive and default mode brain networks. *Proceedings of the National Academy of Sciences* **110**, 18692-18697 (2013).

15. Rogachov A, Cheng JC, Erpelding N, Hemington KS, Crawley AP, Davis KD. Regional brain signal variability: a novel indicator of pain sensitivity and coping. *PAIN* **157**, 2483-2492 (2016).

16. Hemington KS, Wu Q, Kucyi A, Inman RD, Davis KD. Abnormal cross-network functional connectivity in chronic pain and its association with clinical symptoms. *Brain Structure and Function* **221**, 4203-4219 (2016).
