# Supplementary material for: A Hidden Markov Model reveals magnetoencephalography spectral frequency-specific abnormalities of brain state power and phase-coupling in neuropathic pain
Source: Commun Biol. 2022 Sep 21;5:1000. doi: 10.1038/s42003-022-03967-9 (PMC9492713; doi:10.1038/s42003-022-03967-9)
Supplement: Supplementary file 2 — Description of Additional Supplementary Files [file 42003_2022_3967_MOESM2_ESM.pdf]

Description of Additional Supplementary Files

File name: Supplementary Data 1
Description: The source data behind the Fig. 2b-c, 3 and 5 in the paper.
