# Supplementary material for: A Hidden Markov Model reveals magnetoencephalography spectral frequency-specific abnormalities of brain state power and phase-coupling in neuropathic pain
Source: Commun Biol. 2022 Sep 21;5:1000. doi: 10.1038/s42003-022-03967-9 (PMC9492713; doi:10.1038/s42003-022-03967-9)
Supplement: Supplementary file 4 — Reporting Summary [file 42003_2022_3967_MOESM4_ESM.pdf]

nature portfolio

Corresponding author(s): Karen D. Davis

Last updated by author(s): August 31, 2022

Reporting Summary

Nature Portfolio wishes to improve the reproducibility of the work that we publish. This form provides structure for consistency and transparency in reporting. For further information on Nature Portfolio policies, see our Editorial Policies and the Editorial Policy Checklist.

Statistics

For all statistical analyses, confirm that the following items are present in the figure legend, table legend, main text, or Methods section.

n/a	Confirmed	
☐	☒	The exact sample size (*n*) for each experimental group/condition, given as a discrete number and unit of measurement
☐	☒	A statement on whether measurements were taken from distinct samples or whether the same sample was measured repeatedly
☐	☒	The statistical test(s) used AND whether they are one- or two-sided<br>*Only common tests should be described solely by name; describe more complex techniques in the Methods section.*
☐	☒	A description of all covariates tested
☐	☒	A description of any assumptions or corrections, such as tests of normality and adjustment for multiple comparisons
☐	☒	A full description of the statistical parameters including central tendency (e.g. means) or other basic estimates (e.g. regression coefficient) AND variation (e.g. standard deviation) or associated estimates of uncertainty (e.g. confidence intervals)
☐	☒	For null hypothesis testing, the test statistic (e.g. *F*, *t*, *r*) with confidence intervals, effect sizes, degrees of freedom and *P* value noted<br>*Give P values as exact values whenever suitable.*
☒	☐	For Bayesian analysis, information on the choice of priors and Markov chain Monte Carlo settings
☒	☐	For hierarchical and complex designs, identification of the appropriate level for tests and full reporting of outcomes
☐	☒	Estimates of effect sizes (e.g. Cohen's *d*, Pearson's *r*), indicating how they were calculated

Our web collection on statistics for biologists contains articles on many of the points above.

Software and code

Policy information about availability of computer code

Data collection	Standard anatomical MRI and MEG scanner software<br>MRI scan: 3T GE Signa HDx MRI scanner (GE Medical Systems, Milwaukee, WI)<br>MEG scan: 306 channel Elekta Neuromag TRIUX system
Data analysis	Standard imaging tools for preprocessing Fieldtrip (http://www.fieldtriptoolbox.org/) , SPM, FSL(v5.0), and the OHBA Software Library (OSL; https://ohba-analysis.github.io/osl-docs/)<br>MATLAB scripts (5.7.12.0 ; MathWorks) were used for whole-brain modelling and HMM analysis, which are freely available. The HMM is inferred using the HMM-MAR toolbox https://github.com/OHBA-analysis/HMM-MAR, through the hmmmar.m function (details about the options can be found here https://github.com/OHBA-analysis/HMM-MAR/wiki/User-Guide#-hmm-marmodel-estimation)

For manuscripts utilizing custom algorithms or software that are central to the research but not yet described in published literature, software must be made available to editors and reviewers. We strongly encourage code deposition in a community repository (e.g. GitHub). See the Nature Portfolio guidelines for submitting code & software for further information.

Data

Policy information about availability of data

All manuscripts must include a data availability statement. This statement should provide the following information, where applicable:

- Accession codes, unique identifiers, or web links for publicly available datasets
- A description of any restrictions on data availability
- For clinical datasets or third party data, please ensure that the statement adheres to our policy

The code is freely available from github and the data that support the findings of this study are available upon reasonable request from the corresponding author Dr. Karen Davis. The data are not publicly available due to third party restriction and patient privacy issues of the institution.

Human research participants

Policy information about studies involving human research participants and Sex and Gender in Research.

Reporting on sex and gender	Our research group follows the Canadian Institutes of Health Research (CIHR) recommendations for Sex- and Gender Based-Analysis (SGBA; (www.cihr-irsc-igh-isfh.ca/?lang=en). Findings apply for both sexes, and sex factor was considered in the study design (and individuals consented to share this information). The two groups of participants (patients and controls) were age- and sex-matched. Results are controlled for sex, age and disease length when appropriate. However, in the present study, due to the sample size, it was not possible to explicitly test for sex differences within and across groups. Regarding gender, our previous data collection did not include gender self-identification questionnaires and did not document gender-related variables that can be used as proxies (e.g., socioeconomic status, occupation, etc.) in a second level analysis or to create a gender index. Thus, we cannot develop a gender story in our analyses but now plan to include these important queries in our future studies going forward.
Population characteristics	This study included 40 patients diagnosed with neuropathic pain (NP; 20 males (mean age in years ± SD = 41.4 ± 8.3) and 20 females (mean age in years ± SD = 42.7 ± 9.3); range [24-63]) and 40 age- and sex-matched healthy controls (HCs; 20 males (mean age = 40.9 ± 9.6), 20 females (mean age = 41.6 ± 8.1); range [24-59]).<br>The neuropathic pain group consisted of 11 patients with ankylosing spondylitis (5 males), 10 with multiple sclerosis (6 males), 10 with carpel tunnel syndrome (4 males), and 9 with other neuropathic etiologies (5 males).<br>Treatments including medications are reported in supplemental information.
Recruitment	Patients with chronic neuropathic pain were recruited at Toronto Western Hospital (UHN; ON, CA) and St. Michael's Hospital (Toronto, ON, CA). Recruitment for healthy participants was done through advertisements posted at University Health Network hospitals in Toronto, ON, Canada. They received 75$CAD as reimbursement for their participation to the study.
Ethics oversight	All study procedures were approved by the Research Ethics Boards of the University Health Network, and the St. Michael's Hospital. The study was conducted in accordance with the Declaration of Helsinki, and all participants provided informed written consent.

Note that full information on the approval of the study protocol must also be provided in the manuscript.

Field-specific reporting

Please select the one below that is the best fit for your research. If you are not sure, read the appropriate sections before making your selection.

☒ Life sciences ☐ Behavioural & social sciences ☐ Ecological, evolutionary & environmental sciences

For a reference copy of the document with all sections, see nature.com/documents/nr-reporting-summary-flat.pdf

Life sciences study design

All studies must disclose on these points even when the disclosure is negative.

Sample size	We used data of 80 participants from our database. This sample size is equivalent (or higher) than previous studies using the methodological approach (e.g., Vidaurre, D., Hunt, L.T., Quinn, A.J. et al. Spontaneous cortical activity transiently organises into frequency specific phase-coupling networks. Nat Commun 9, 2987 (2018). https://doi.org/10.1038/s41467-018-05316-z)
Data exclusions	N/A
Replication	N/A : No other publicly available dataset including MEG recordings from patients with chronic neuropathic pain is currently available to test the reproducibility of the findings. However, our results are well consistent with previous studies in other populations (e.g., Vidaurre et al 2018; Quinn et al., 2018).
Randomization	N/A

Blinding	N/A

Reporting for specific materials, systems and methods

We require information from authors about some types of materials, experimental systems and methods used in many studies. Here, indicate whether each material, system or method listed is relevant to your study. If you are not sure if a list item applies to your research, read the appropriate section before selecting a response.

Materials & experimental systems

n/a	Involved in the study
☒	☐ Antibodies
☒	☐ Eukaryotic cell lines
☒	☐ Palaeontology and archaeology
☒	☐ Animals and other organisms
☒	☐ Clinical data
☒	☐ Dual use research of concern

Methods

n/a	Involved in the study
☒	☐ ChIP-seq
☒	☐ Flow cytometry
☒	☐ MRI-based neuroimaging

March 2021
